# An Interdisciplinary Definition of Pornography: Results from a Global Delphi Panel

**DOI:** 10.1007/s10508-019-01554-4

**Published:** 2019-09-23

**Authors:** Alan McKee, Paul Byron, Katerina Litsou, Roger Ingham

**Affiliations:** 1 Faculty of Arts and Social Sciences, University of Technology Sydney, Ultimo, NSW Australia; 2 Centre for Sexual Heath Research, School of Psychology, University of Southampton, Southampton, SO17 1BJ UK

**Keywords:** Pornography, Definitions, Sexually explicit material, Interdisciplinary research

## Abstract

In interdisciplinary investigations into the relationships between pornography and its audiences, the issue of how to define the object of study is more complex than in studies situated within a single discipline. A Delphi panel of 38 leading pornography researchers from a wide range of disciplines was asked about various topics, including the definition of pornography. Qualitative and quantitative analyses of two rounds of survey responses suggested two different and—at first sight—incompatible definitions operating. The first was “Sexually explicit materials intended to arouse.” The second was a culturally relative definition suggesting pornography has no innate characteristics. This technical report suggests that we should encourage researchers to choose which definition they want to use in a self-reflective way depending on the needs of the project, so long as they make it explicit and justify their decision.

## Introduction

This technical report presents the results of an international Delphi panel of 38 leading pornography researchers from a range of disciplines to seek their advice on definitional matters for a project that seeks to understand why different disciplines have produced irreconcilable data about the relationship(s) between pornography and consumers (McKee & Ingham, 2018). In this technical report, we focus on definitions of pornography, paying particular attention to the question of whether it is possible to reach consensus on a definition across disciplines.

“[T]he future of research is increasingly interdisciplinary” (Bridle, Vrieling, Cardillo, & Araya, 2013) and, in order to address complex real-world problems such as the relationships between the consumption/use/exposure to (different disciplines use different terms for this relationship) pornography and aspects of healthy sexual development, sexual health researchers must engage in interdisciplinary work. The rise of smartphones, allowing easy private access to sexually explicit material, especially for young people, has made these issues particularly pressing for many researchers. Some of the research in this area takes the form of interdisciplinarity between closely cognate disciplines which share epistemological assumptions, data gathering methods, and language (e.g., public health and health communication). Although this makes the design and practice of projects simpler, it also serves to avoid the potential richness and attention to complexity that can emerge from projects where members of a research team come from disciplines that have different understandings of these issues (McKee & Ingham, 2018). Although researchers have sought to understand the relationships between the consumption/use/exposure to pornography for many decades—and this has constituted a formal project for academic researchers since the US President’s Commission on Obscenity and Pornography commissioned a number of studies in this area (Wilson, 1973)—it is only recently that researchers have sought to operationalize a definition of pornography for use in academic research (Willoughby & Busby, 2016).

In the discipline of psychology, agreed definitions are taken to be cornerstones for the development of formal theories (Sell, 2018) and psychology researchers have begun to approach a consensus about how to define pornography (sometimes the term sexually explicit material is used as a synonym [Downing, Schrimshaw, Antebi, & Siegel, 2014]), employing definitions that focus on two elements. The first is that pornography is “explicit” (Wright & Randall, 2012) and includes “images of exposed genitals and/or depictions of sexual behaviors” (Morgan, 2011) that are “unconcealed” (Peter & Valkenburg, 2011). The second is that pornography is “intended to increase sexual arousal” (Morgan, 2011).

Even within the discipline of psychology, however, there is little agreement about elements of this definition. Some researchers will include in their definition of pornography texts that show only “nudity” with no sexual contact (Wright & Randall, 2012) (in this technical report, we use the word “texts” in the sense in which it is used by cultural studies researchers—that is, any element of culture that carries meaning for a consumer. This can include books, films, and photographs as well as T-shirts, coffee mugs, or even hairstyles, to name only a few possibilities) (McKee, 2003). By contrast, other researchers in psychology insist that in order to be explicit pornographic texts must show sexual acts or “(aroused) genitals” (Peter & Valkenburg, 2011)—researchers in the latter group exclude *Playboy* from their definition of pornography, for example (Træen & Daneback, 2013). In relation to the second part of the definition, some psychology researchers exclude the intention to arouse and include all sexually explicit materials in their definition of pornography (Træen & Daneback, 2013; Wright & Randall, 2012). And it is notable that even in recent work many researchers do not provide a definition of pornography at all (Doonwaard, van den Eijnden, Overbeek, & ter Bogt, 2015; Downing et al., 2014; Hald, Kuyper, Adam, & de Wit, 2013).

Some researchers within the discipline of psychology have offered more fundamental challenges to the definition. Willoughby and Busby (2016) surveyed 2089 individuals sampled from the Amazon Mechanical Turk (MTurk) Web site, asking them which of a series of types of texts—such as “An image of a woman alone posing in a suggestive way without any clothing on” or “A major Hollywood film or movie that includes one graphic sexual encounter” they would define as pornography. Their “most important finding” was “the large variability that existed in the responses to the items… it was found that items viewed as the most and least pornographic loaded together well but items in the middle of the spectrum exhibited significant variation in responses” (p. 683). They also found that what people perceived as pornographic material was significantly related to usage patterns, where the participants who rated more contents as pornographic appeared to be using such pornographic materials more (Willoughby, Busby, & Young-Petersen, 2018).

Given the lack of consensus within a single discipline, it is not surprising that, when we begin to consider other disciplines that are interested in the consumption/use/exposure to pornography, there are even more pronounced disagreements. Researchers in humanities disciplines insist on the heterogeneity of the category and the variety of texts that can function as pornography, including the complicated relationship the category has with others, including art and sex education. They point out that texts that are produced for other purposes are used for pornographic purposes—such as shoe catalogues used as masturbatory aids by foot fetishists (Rose, 2012). For humanities researchers, the practice of pornography consumption/use/exposure will always exceed a strict definition of what is or isn’t pornography:

The dominant definitions cannot handle such multiplicity, for pornography, the dominant social concepts, cannot possibly cover pornography, the actual aesthetic forms. (Andrews, 2012, pp. 458-459)

It is perhaps not surprising that academics across a range of disciplines have produced a series of definitions that ultimately appear to have little in common:

Pornography is variably defined as the production of sexual representation for the purpose of exchange (Huer); artistic material with little, if any, aesthetic value (Berger); the representation of persons as mere sexual objects (McElroy); [or] the representation of institutional inequality between the sexes (Dworkin; MacKinnon; Langton). (Rose, 2012, p. 458)

Indeed, an influential account of pornography by literary historian Kendrick (1996) insists that pornography is “not a thing but a concept, a thought structure”; different cultures at different times categorize different texts as pornographic as a way to control forms of knowledge and thus power relations between groups. For Kendrick, pornography does not have “*any* common qualities” (Williams, 1989, italics in original).

Based on the current literature, particularly when we take an interdisciplinary perspective, it is difficult to determine common features that would be acceptable to researchers in multiple disciplines as necessary elements of a definition of pornography.

## Method

The project from which the data in this technical report are drawn aims to take an interdisciplinary approach to understanding the relationship between the consumption/use/exposure to pornography and aspects of healthy sexual development by means of a series of extensive systematic literature reviews across social science and humanities disciplines. In order to establish measures and definitions for such a project, the first step was to conduct an interdisciplinary Delphi panel of leading pornography researchers across the range of humanities and social science disciplines that have produced knowledge about this topic. Members were invited via recommendation from an Advisory Group comprising six leading senior academics from a range of disciplines across the social sciences and humanities chosen for their expertise in healthy sexual development and/or representations of sexuality. This included researchers positioned in sexual development, diversity and health, adolescent medicine, sexual and reproductive health, cultural studies, and feminist media studies. They were asked to provide names of “key pornography researchers around the world” to be part of the Delphi panel; between them, they suggested 57 different researchers.

We contacted each of those researchers and invited them to take part. Forty-nine responded, 44 said they would take part, and 38 completed the survey. The resulting panel included experts from a wide range of disciplines, including psychology, communication studies, cultural studies, media studies, human geography, history, literary studies, film studies, gender studies, cultural anthropology, sociology, and public health. A survey instrument was developed containing questions about the relationship between the consumption/use/exposure to pornography and aspects of healthy sexual development; this included asking for a definition of pornography among other topics. The instrument was reviewed by the Advisory Group and revised based on their suggestions.

In the first round of the panel survey, participants were asked to provide an open-ended definition of pornography among other questions about the relationship between the consumption/use/exposure to pornography and healthy sexual development. They were also asked to indicate both the disciplines in which they conducted their doctoral research, and to self-nominate their current research discipline. Analysis of the results and discussion between the authors noted that there appeared to be two groups of responses about the definition of pornography. Accordingly, a second survey of the Delphi panel members was conducted, offering these two possible alternative definitions of pornography and asking the panelists to rate their level of agreement with them on a Likert scale. It should be noted that these two definitions appear, at first sight, to be incompatible. All 44 Delphi panel members who enrolled in the study (including those who did not complete the first survey) were invited to participate. Twenty-seven respondents completed this tranche of the survey.

## Results

In the first round of the Delphi panel, we asked respondents “How would you define pornography?”; 36 members of the survey panel answered the question. No two researchers gave exactly the same definition. Over three-quarters of the 36 respondents who attempted this task included the term “explicit” within their definition; for example:

“sexually explicit media”; “the explicit representation of sexual activity, broadly defined, in images and words”; “sexually explicit materials within different media and art formats”; “the public presentation of actually occurring sexual relations.”

Just over half included “intention to arouse” or similar phrase; examples include:

“material designed to provide arousal and entertainment of a sexual nature”; “material that is designed to provoke urges to masturbate”; “an aesthetic work with the primary artistic intention to encourage sexual arousal or other forms of autoerotica”;“explicit sexual representation for the purpose of arousal.”

Over half of the sample mentioned only one term or the other, and less than half included both these terms (or cognates) in their definitions. Some of the participants who offered a definition including both elements (or cognates) also added *caveats* to their definition; these included the use of qualifiers such as “porn often contains” or the use of “and/or” to link explicitness and arousal, or providing alternative definitions alongside this one; for example: “Pornography can be understood in two ways: (1) …sexual explicitness and/or a purposive attempt to arouse… (2) …a frequently disparaging label applied to media texts possessive of a particular set of characteristics such as affectivity, transgressiveness and prurience.”

Researchers disagreed on the place of “intent” in the definition. Some did state that pornographic material must be “designed,” “produced,” “for the purpose of” or “aiming at” or “intended” to or “meant” to provide arousal. However, other researchers suggested that material is pornographic if it is “consumed” for sexual arousal, or “stimulates,” or “sexually arouses.”

Another group of respondents did not use the language of explicitness or intent to arouse, but instead presented a different kind of definition; they insisted that pornography is not a “thing” but an “argument” or a “process.” The six respondents in the latter camp comprised five who nominated either their doctoral degree or current area of study as film studies or media studies, and one historian. Two mentioned the genre theories of Altman, whereby the content of a genre is the result of a “contract” between producers and “a community of users—audiences, fans, critics, etc.”; for example:

I tend to consider pornography as an audiovisual genre; therefore, I’d extend to pornography the semantic-syntactic-pragmatic approach developed by Rick Altman. In this sense, I consider pornography as a complex set of semantic elements (for instance, but not exclusively, explicit sex), syntactic elements (specific plot structures, or visual styles, etc.) and pragmatic elements (in this case, the existence of a community of users—audiences, fans, critics, etc.—that considers a specific object as pornographic).

Two mentioned Kendrick’s history of pornography, which argues that pornography is an “argument” or a “process, not a thing” as illustrated in the following:

I would hesitate to define it, suggesting as Walter Kendrick does that “pornography” names an argument, not a thing.

I tend to go with (and expand) Walter Kendrick’s definition: Pornography is a process, not a thing. That process involves cultural shifts, norms, regulations, social relations, taboos, and sanctioned/unsanctioned pleasures and desires.

To gloss this last response, definitions of pornography “change over time” and the decision about who gets to decide what counts as pornographic and what they count as pornography is tied up with social and cultural relations of power. (In Kendrick’s example, it was unproblematic for educated rich white men in the nineteenth century to view erotica, but when it became widely available through cheap printing to the uneducated masses it became “pornography” and had to be controlled.)

So, two distinct approaches to the definitions of pornography were identified; the first is “sexually explicit materials intended to arouse,” implying an essence to pornography—all pornographic texts will have similar characteristics under this definition. The second approach is culturally relative—it states that at a given time in a given culture, there will be rules about what is and what is not pornographic, but that these rules can change. At some points in time, the category “pornography” will include only sexually explicit materials intended to arouse, but, at other times, other kinds of texts will be included in the category of pornography and “sexually explicit texts intended to arouse” may not be captured in the category. Arguments about which texts should be included in the category of pornography become power struggles—as we can see in fights about, for example, whether sex education textbooks (McKee, 2017) or artworks (Simpson, 2011) are pornographic.

On the basis of these responses, we identified two (what we thought would be) incompatible themes in the definitions of pornography offered by researchers; these were: (1) Sexually explicit materials intended to arouse, and (2) Pornography is not a thing but a concept, a category of texts managed by institutions led by powerful groups in society in order to control the circulation of knowledge and culture, changing according to geographical location and period.

In the follow-up Delphi panel survey, we asked researchers for their level of agreement with each of these two definitions of pornography (using 5-point Likert scales from 1 = Strongly Disagree to 5 = Strongly Agree). Note that by using five-point scales, as opposed to a simple binary agree/disagree response mode, participants were able to indicate partial agreement; for example, if they agreed with some aspect(s) of the definition but not all of them. There was also an “It depends” option with a request to elaborate, but none of the participants made use of this in respond to these questions.

Of the 27 participants who replied to this tranche of the survey, 21 Agreed or Strongly Agreed with the first definition *Sexually explicit materials intended to arouse*, while just two Disagreed or Strongly Disagreed. For the second definition, 15 respondents Agreed or Strongly Agreed, while nine Disagreed or Strongly disagreed.

Using a value of 1 for Strongly Disagree through to 5 for Strongly Agree, the mean scores for the two definitions were 4.19 for definition 1 and 3.50 for definition 2. Data were divided by the disciplinary background of the participants. The mean levels of agreement for definition 1 (material intended to arouse) were 4.30 for social scientists and 2.70 for humanities researchers. Corresponding figures for definition 2 (not a thing but a concept) were 4.13 and 4.00, respectively. In other words, social scientists were more likely to agree with both definitions (4.30 and 4.13, respectively), whereas humanities researchers were more inclined to agree with definition 2 (4.00) than with definition 1 (2.70). Further exploration of the ratings revealed that just over half of the 27 participants Agreed or Strongly Agreed with both definitions.

## Discussion

The fact that researchers across disciplines did not agree on a single definition of pornography is both unsurprising and surprising. It is unsurprising in that several recent researchers have made the same point (Andrews, 2012; Rose, 2012). But it remains a surprising finding because researchers have been gathering data about the relationships between pornography and its consumers/users/those exposed to it for several decades (Sullivan & McKee, 2015); given this, the fact that there is not yet an agreed definition of the focus of study is surprising.

The results of the first round of the survey show that more than half of the surveyed researchers used each of the terms “explicit” and “arouse,” which might offer some hope that “Sexually explicit content intended to arouse” could offer a starting point for a consensus on definition. But as we note, only a minority of panelists used both of these terms without caveats. We also note the disagreement among researchers as to whether pornographic material must be created with the intent to arouse or whether it is defined by the fact it is consumed to create arousal. The complexity of the different definitions that can be created through different applications of explicitness and/or arousal (with the latter term having two possible meanings—intent to arouse or use for arousal) leads to a complex matrix of definitions, each of which produces a different object of study. One respondent mentioned both explicitness and intent to arouse—and then noted that under their definition this would exclude *Playboy*, as it is does not contain “clear and explicit acts.” Another mentioned only that material must be explicit, and not that it be designed for sexual arousal—which could include sex education materials. Another respondent included material designed for sexual arousal, even if not explicit—which could include romance novels, for example. One excluded both explicitness and intent, defining pornography as “Any material…that sexually arouses people,” which, they note, would include some of the pictures in *National Geographic* among other materials that are not produced for masturbatory purposes.

The fact that some researchers defined pornography in terms of textual qualities while others insisted that pornography is “an argument” or “a process, not a thing” points to a fundamental disagreement between disciplines in defining pornography. The question of whether *pornographicness* (to coin an ugly neologism) is a quality that is possessed by texts, or a quality that is assigned to particular texts at particular times by particular institutions and discourses, is not one that will be easily answered. That is to say, can something that is pornography in a particular culture at a particular time stop being pornography in a different culture or different time, and vice versa? It is perhaps not surprising that the film/media studies researchers tended to favor definitions that were culturally—and temporally—specific, while the psychologists tended to assign pornographicness to the text itself. It is a cliché of humanities researchers that their answer to any straight question is “it depends”; and so it was a humanities researcher who started their definition of pornography with the phrase “The question—as the researchers are well aware—is an extremely fraught one.”

Responses to the second tranche of the survey further confused the issue. As noted above, the two definitions we tested were, to some extent, incompatible. Nevertheless, 14 out of 27 respondents either Agreed or Strongly Agreed with both. Only seven respondents Agreed or Strongly Agreed with one definition, and Disagreed or Strongly Disagreed with the other, all of whom Agreed or Strongly Agreed with the first definition—“Sexually explicit materials intended to arouse”—and Disagreed or Strongly Disagreed with the second definition (“not a thing but a concept”). Of these seven respondents, four were in the social sciences (psychology, communication studies, and public health), while three were in the humanities (media studies and cultural studies). Just one respondent Disagreed or Strongly Disagreed with both definitions (a humanities researcher from film studies).

This complex pattern of responses suggests that, rather than a single definition of pornography, if we employ *both* definitions we are more likely to meet the needs of an interdisciplinary cohort of pornography researchers. But how could a dual definition be operationalized in future research?

As noted above, for some researchers the complexity of the reality of pornography must elude any possible definition in language. To say that pornography is “sexually explicit material intended to arouse” may not, as Willoughby and Busby (2016) argue, match up with the ways in which consumers are deciding what pornography is in their own consumption practices. As Andrews (2012, p. 459) writes, “pornography, the dominant social concepts, cannot possibly cover pornography, the actual aesthetic forms.” For researchers taking this perspective, the second definition fits their approach:

Pornography is not a thing but a concept, a category of texts managed by institutions led by powerful groups in society in order to control the circulation of knowledge and culture, changing according to geographical location and period.

This definition works well for projects that do not need to gather empirical data; projects in those disciplines where the very act of keeping open the discussion of how to define pornography is the very point of the work itself. For example, Rose’s (2012) philosophical article “The definition of pornography and avoiding normative silliness: a commentary adjunct to Rea’s definition” produces a definition of pornography, only to then critique an element of his own definition as being problematic, before then noting that “such a metaphysics of community is beyond the scope of this paper,” and ending the argument there (Rose, 2012). Researchers in these disciplines can refuse to compromise on an imperfect definition of pornography, always pointing out problems with any proposed definition, thus infinitely delaying a final consensus. This approach remains true to lived complexity, but fails to produce final, agreed operational terms that allow for empirical research. Humanities researchers are fascinated by the definition of terms and discussing definitions of key terms is a vital part of the humanities project; but there is little sense that at any point a definition must be locked down and agreed so that discussions about it can end.

However, the relationship to definitions of researchers working on empirical projects must be slightly different. If one wishes to conduct empirical research to produce replicable data—such as through surveys and content analysis—it is necessary to agree on a definition of the object being studied, even if that definition is understood to be imperfect or incomplete.

We might then propose that it is possible to operationalize two incompatible definitions of pornography for academic research by saying that the definition a researcher chooses should depend on the nature of the project they are running, and the purposes to which they need to put the definition. Researchers in disciplines such as history, cultural studies or literary studies may wish to insist that pornography is no single thing, and so, for them, there is no driving need to stop discussing the definition of the term. Meanwhile, for researchers in psychology, for example, it may be necessary to reach a single, agreed, operational definition in order to allow them to gather data and map relations between variables to be explored. This would represent a pragmatic approach to definition(s). We know very well that there is no single, homogenous group of texts that constitutes the genre “pornography”; we know from historical and cross-cultural research that “pornography” changes from country to country and era to era depending on the institutions managed by powerful groups for their own ends…but all the same it makes (a certain kind of) sense to describe pornography, at this time, in Western countries, as “sexually explicit texts intended to arouse.” We can use this definition, with certainty, to gather data about how certain texts circulate and function in our current countries—so long as we always bear in mind that this definition is always provisional, will change over time—and, crucially, we maintain a self-awareness about the fact that this definition itself suits the purposes of powerful groups who wish to control the circulation of knowledge and culture in our societies.

Indeed, we think we can see some evidence in our Delphi panel responses that many researchers are already, in practice, balancing a tension between complexity and operational necessity. Twenty-eight panelists provided a definition of pornography that included the terms “explicit” and/or “intended to arouse” (or cognates). In a later question, panelists were asked “In your professional opinion, what is the relationship of pornography with its audiences/users/consumers etc.?” In response to this question, 20 of these respondents—despite presenting a workable operational definition of pornography—included terms that insisted on complexity and variability, such as “It’s many things,” “That really does depend entirely on the circumstances,” “Depends on the type of material,” “Very complicated!,” “Too diverse to sum up neatly,” “Complex, and contingent upon many factors,” “It depends,” and “multiple, diverse.” For example, one researcher who defined pornography as “sexually explicit visual or printed material that is consumed for sexual arousal” also stated that the relationship between pornography and its consumers is “Too diverse to sum up neatly, there are so many different kinds of pornography and so many different consumers!”. In each of these cases, a researcher is aware of the fact that a simple definition of pornography cannot do justice to the complexity of its reality, but is willing to make a contingent decision to lay out a simple definition in order to allow empirical data gathering to take place. And, by settling on a definition, researchers also increase the possibility of communication with other researchers who may work within other disciplines—even if that communication takes the form of disagreement, at least there is something to talk about.

Although the members in this panel were identified by our Advisory Group as being experts in the field of pornography research, Baker, Lovell, and Harris (2006) have noted that there is no agreed definition of the term “expert” in methodological writing about Delphi panels. Further, Akins, Tolson, and Cole (2005) note that there is no agreed number of experts for a Delphi panel to guarantee stability of results. Hsu and Sandford (2007) note that, in designing a Delphi instrument, researchers must be careful not to lead the respondents. The authors do not claim that the Delphi panel consulted represents all experts in pornography research globally. A sample of different experts might produce different results, particularly given the interdisciplinary nature of the cohort.

As researchers increasingly practice interdisciplinary investigations into real-world problems, including the relationships between pornography and its audiences, the issue of how to define the object of study will only become more complex. The data from these Delphi panel surveys show that different disciplines have different approaches to thinking about the nature of pornography and how that might be captured in language. The results of these surveys suggest that one way forward might be to apply two incompatible definitions of pornography—one which says that pornographicness is a quality of texts, one saying it is produced by cultural context—and to allow researchers to choose which they want to use in a self-reflective way depending on the needs of the project, so long as they can make explicit and justify their decision.
